# Health-Related Quality of Life of Persons with Direct, Indirect and No Migration Background in Germany: A Cross-Sectional Study Based on the German Socio-Economic Panel (SOEP)

**DOI:** 10.3390/ijerph18073665

**Published:** 2021-04-01

**Authors:** Thomas Grochtdreis, Hans-Helmut König, Judith Dams

**Affiliations:** Department of Health Economics and Health Services Research, Hamburg Center for Health Economics, University Medical Center Hamburg-Eppendorf, Martinistr. 52, 20246 Hamburg, Germany; h.koenig@uke.de (H.-H.K.); j.dams@uke.de (J.D.)

**Keywords:** SF-12, surveys and questionnaires, health, quality of life, migrant

## Abstract

Global migration towards and within Europe remains high, shaping the structure of populations. Approximately 24% of the total German population had a migration background in 2017. The aim of the study was to analyze the association between migration background and health-related quality of life (HrQoL) in Germany. The analyses were based on 2014 and 2016 data of the German Socio-Economic Panel. Differences in sociodemographic characteristics between migrant and non-migrant samples were equal by employment of the entropy balancing weights. HrQoL was measured using the physical (PCS) and mental (MCS) component summary scores of the SF-12v2. Associations between PCS and MCS scores and migration background were examined using Student’s *t*-test. The mean PCS and MCS scores of persons with migration background (n = 8533) were 51.5 and 50.9, respectively. Persons with direct migration background had a lower PCS score (−0.55, *p* < 0.001) and a higher MCS score (+1.08, *p* < 0.001) than persons without migration background. Persons with direct migration background differed with respect to both physical and mental HrQoL from persons without migration background in the German population. Differences in HrQoL for persons with indirect migration background had *p* = 0.305 and *p* = 0.072, respectively. Causalities behind the association between direct migration background and HrQoL are to be determined.

## 1. Introduction

In Germany, about 19.3 million persons had a migration background in the year 2017, corresponding to a proportion of about 23.6% of the total German population [1]. By definition of the German Institute for Economic Research (DIW Berlin), persons with a direct migration background are persons with their own migration experience born without German citizenship, and persons with an indirect migration background are persons without their own migration experience who were born to at least one parent with direct migration background [2]. This definition is concurrent with the definition of migrants and second-generation migrants by the European Migration Network [3,4]. In general, the spectrum of migration to Germany and the histories of persons with migration background have changed in the past decades, inter alia, through the increasing globalization and through the generations of persons growing up who were born to parents who became sedentary in Germany after passing through the status of guest worker [5,6]. In the 1990s and 2000s, with the fall of the Iron Curtain, the development of new economic flows between European regions, the emergence of new areas of origin, and the entry into the European Union of 12 countries, new migratory flows have emerged in Europe [7].

As the proportion of persons with migration background is increasing throughout Europe, there is also a need for monitoring and extending knowledge on migrants’ health and quality of life, not least for the provision of adequate and accessible health care services [8,9]. Reduced health and quality of life might mutually affect engagement in education, work, and social activities and thus have an influence on the integration of persons with migration background as a whole [8]. Reasons for a reduced health and quality of life might be informal barriers to accessing health care, such as a complex interaction between language, communication, and sociocultural factors, but also an interlink with migration and ethno-cultural diversity [8,10,11]. However, the influence of migration, especially for the new generation of persons with migration background and for persons with indirect migration background, is not yet fully resolved.

Earlier studies on health and quality of life of persons with migration background in Germany mainly focused on the healthy-migrant effect and the health and quality of life of migrant workers [12,13,14,15], whereas one recent study focused on the trajectories of health-related quality of life (HrQoL) in persons with and without migration background in Germany [16]. In further studies that analyzed the association of HrQoL with migration background in Germany, no association with physical HrQoL has been found, whereas mental HrQoL was negatively associated with migration background [14,16,17]. Indeed, persons with migration background are commonly known to be comparatively healthy, but yet this might not be true for recently migrated persons and persons with indirect migration background, as the sociodemographic diversity of persons with migration background and particular health challenges might be not the same [18]. Also, persons with indirect migration background might have greater health challenges than persons with direct migration background [18,19,20].

However, not much is known about potential differences in HrQoL of those persons who migrated during the new migratory flows that have emerged in Europe and those persons who are descendants of parents with direct migration background who became sedentary in Germany compared to those persons without migration background. Knowledge about differences in HrQoL between different person groups is particularly of interest for research and policy-makers in order to be able to focus on possible target-specific healthcare services and clinical implications. Therefore, it is necessary to refocus research on health and quality of life of persons with migration background, with a special focus on those persons with direct migration background that had migrated in the 1990s and 2000s as well as on those persons with indirect migration background. Our hypothesis is that, in contrast to the positive effects of migration on HrQoL that have been found for the generation of migrant workers [12,13,14,15], the migration background of the more recently migrated persons and those persons with indirect migration background is negatively associated with HrQoL. The aim of this study, therefore, was to analyze the associations between direct, indirect, and no migration background and the physical and mental HrQoL of persons in Germany.

## 2. Materials and Methods

### 2.1. Sample

The sample of this study was based on cross-sectional data of the German Socio-Economic Panel (SOEP), provided by the German Institute for Economic Research (DIW Berlin). The SOEP is a representative German household panel with over 20,000 participants annually since 1984, with 35 waves available by 2019. In order to ensure that the previously underrepresented current generation of persons with migration background was represented in the SOEP proportionally to their share of the German population, two additional migrant samples (M1 and M2) were integrated into the SOEP [21].

**Figure 1:**
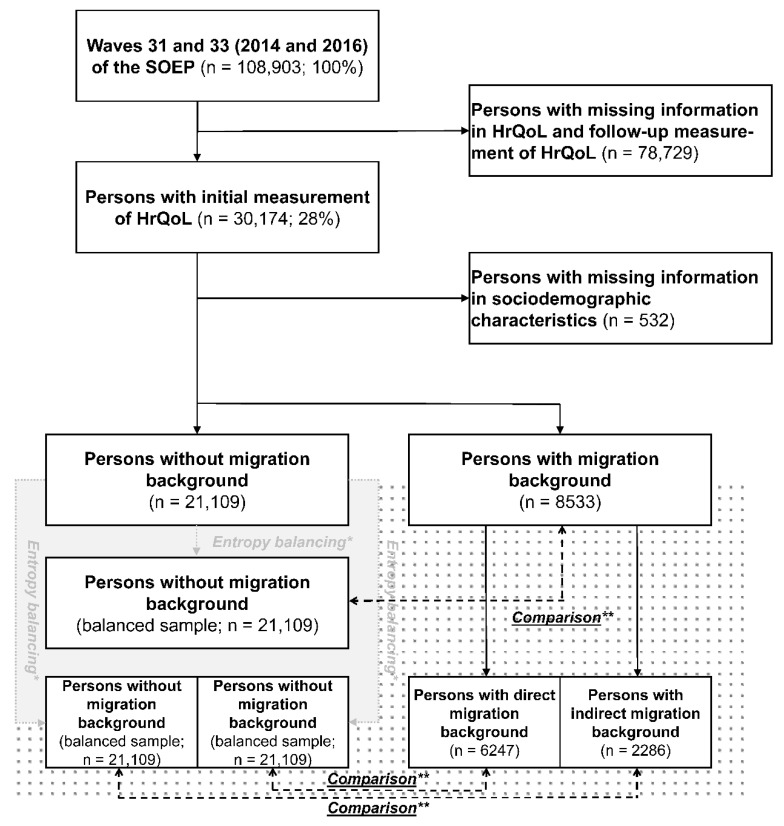
Flow chart of the selection and reweighting process. SOEP: German Socio-Economic Panel, HrQoL: health-related quality of life; * For the sample of persons without migration background, three different weights were derived using entropy balancing, thus differences in means and standard errors of sociodemographic characteristics between persons with and without migration background, persons with direct and without migration background, and persons with indirect and without migration background were equal by employment of the entropy balancing weights in the explanatory models. ** Differences in HrQoL with respect to migration background, direct migration background, and indirect migration background were evaluated by comparison with the respectively reweighted sample of persons without migration background.

For the samples M1 and M2, households of persons who had immigrated to Germany during the years 1994 to 2009 and 2010 to 2013 were selected, respectively. By 2019, five waves of the M1 sample and three waves of the M2 sample were available (waves 30 to 34). However, as HrQoL was surveyed only in even years since the year 2002, data sets from the waves 31 and 33 (2014 and 2016) were used (n = 108,903; 100%). Out of these waves, a sample was generated by removing persons with missing information in HrQoL and by using only the initial measurement of HrQoL in the respective wave in order to avoid interdependence of repeated measurements (n = 30,174; 28%). Furthermore, persons with missing information in sociodemographic characteristics were removed, resulting in a net sample of n = 29,642 (27%). A flow chart of the selection process is presented in Figure 1.

### 2.2. Measures

In order to distinguish between persons with direct and indirect migration background and persons without migration background, a predefined SOEP variable was used. In consistence with the definition of direct and indirect migration background of the DIW Berlin, this variable combined information on country of birth, citizenship, and parental information to derive whether a person had an own migration experience or was born to at least one parent with direct migration background [2]. Persons born in another country than Germany were assigned a direct migration background, and persons born in Germany whose father and/or mother had a migration background were assigned an indirect migration background. Persons born in Germany without parents with migration background were assigned no migration background. Furthermore, it was distinguished between persons with and without migration background, whereby persons with migration background consisted of persons with direct and indirect migration background.

In order to measure HrQoL, a modified version of the standardized questionnaire SF-12v2 was used in the SOEP [22,23]. The SF-12 consists of 12 items with 8 subscales: physical functioning, physical role limitations, bodily pain, general health, vitality, social functioning, emotional role limitations, and mental health [24]. In the SOEP, the SF-12 question about ‘work interference due to pain’ was removed, whereas one additional SF-36 question about ‘severe physical pain’ was included. Furthermore, the SF-12 questionnaire in the SOEP deviates to some extent in the layout and in the form and order of the questions [23]. The eight subscales of the SF-12 were Z-transformed by norm-based scoring using mean values and standard deviations of a German normative sample [22,25]. According to Ware et al. [24], a physical component summary (PCS) score was calculated by combining the items of the dimensions physical functioning, role limitations, social functioning, and pain. Furthermore, by combining the items of the dimensions social functioning, emotional role limitations, and mental health, a mental component summary (MCS) score was calculated. Thus, the PCS and the MCS scores represent physical and mental HrQoL on scales ranging between 0 and 100 (with higher scores representing better HrQoL), respectively.

The sociodemographic characteristics age (18–29, 30–39, 40–49, and ≥50), sex (female and male), marital status (never married/single, married/in partnership, separated/divorced, and widowed), and employment status (employed fulltime, employed part-time, apprenticeship, marginally employed, and unemployed) were derived from the SOEP. Furthermore, nationality was categorized into German, East European, South European, West and North European, African, Asian, and American/Oceanian countries of origin according to the geographic regions of the Standard Country or Area Codes for Statistical Use (M49) of the United Nations [26].

### 2.3. Statistical Analysis

In order to evaluate the differences in HrQoL with respect to migration background, direct and indirect migration background, and no migration background, the non-migrant sample was preprocessed in such a way that all samples were balanced with respect to the sociodemographic characteristics. Therefore, three different weights were derived for the non-migrant sample using entropy balancing with the predictors age, sex, marital status, and employment status on the basis of the three migrant (sub-)samples. In the subsequent explanatory models, differences in means and standard errors of those sociodemographic characteristics between persons with and without migration background, persons with direct and without migration background, and persons with indirect and without migration background were equal by employment of the entropy balancing weights [27]. The respective data of persons with migration background, direct and indirect migration background, were used as reference and remained unchanged. A flow chart of the reweighting process is presented in Figure 1.

Descriptive statistics of sociodemographic variables were calculated for the unbalanced samples. PCS and MCS scores were calculated by sociodemographic characteristics for the migrant samples and the balanced non-migrant samples. Furthermore, differences in PCS and MCS scores between persons without migration background and persons with direct and indirect migration background were calculated by sociodemographic characteristics. Differences in PCS and MCS scores by migration background were analyzed using Student’s *t*-test. Weights derived by entropy balancing were included for adjustment of differences in sociodemographic characteristics between persons without migration background and persons with direct and indirect migration background.

All analyses were performed using Stata/SE 16.1 (StataCorp, College Station, TX, USA). Entropy balancing was performed using the Stata command ‘ebalance’ [28]. All applied statistics were two-sided. In total, 13 tests for statistical significance of group differences mean PCS/MCS scores were conducted per sample. Therefore, the level of significance was set at α = 0.004 (0.05/13) to correct for multiple significance tests to avoid a type I error [29].

## 3. Results

### 3.1. Sample Characteristics

Before entropy balancing, persons with migration background (n = 8533) differed in age, marital status, employment status (all with *p* < 0.001) compared with persons without migration background (n = 21,109), whereas no difference in sex was observed (both 54%; *p* = 0.844). With a mean age of 39 years (42 years/29 years), persons with (direct/indirect) migration background were younger than persons without migration background, who were on average 50 years old (*p* < 0.001). Furthermore, the majority of persons with migration background had a German nationality (53.4%), followed by 15.6% and 13.4% with a nationality from a Southern European country and an Eastern European country, respectively. The sociodemographic characteristics of the sample pre-balancing are shown in Table 1.

After balancing, the migrant samples were similar to the non-migrant samples with respect to sociodemographic characteristics. The majority of the total sample was female (53.7%) and was either employed fulltime (37.6%) or unemployed (36.7%). Furthermore, 59.0% were married or in a partnership, 30.9% had never been married or were single, and 8.1% were separated or divorced.

### 3.2. Differences in PCS Scores between Persons with and without Migration Background

The difference in PCS scores between persons with and without migration background had *p* = 0.009 (51.5 vs. 51.9; Table 2). However, women with migration background had lower PCS scores than women without migration background (51.0 vs. 51.6, *p* < 0.001). Persons with migration background aged 50 years and older had a lower PCS score than persons without migration background of the same age (44.2 vs. 46.0, *p* < 0.001). Furthermore, persons being married or in a partnership with migration background had a higher PCS score than persons without migration background with the same marital status (50.4 vs. 51.1, *p* < 0.001).

### 3.3. Differences in MCS Scores between Persons with and without Migration Background

The mean MCS score of persons without migration background was 50.0 (Table 2). The mean MCS score of persons with migration background was higher (50.9, *p* < 0.001). Both women and men with migration background had higher MCS scores than women and men without migration background (49.8 vs. 48.9 and 52.3 vs. 51.2, both with *p* < 0.001). Furthermore, the MCS scores of persons aged less than 50 years was higher for persons with migration background compared with persons without migration background. Thereby, the MCS scores of persons with migration background decreased with higher age (51.0 to 50.6), whereas for persons without migration background, MCS scores increased with higher age (49.6 to 49.9, all with *p* < 0.001). Persons being married or in a partnership as well as never married or single with migration background had a higher MCS score than persons without migration background with the same marital status (50.7 vs. 49.6 and 51.3 vs. 50.7, all with *p* < 0.001).

### 3.4. Differences in PCS and MCS Scores between Persons with Direct/Indirect and without Migration Background

Persons with direct migration background (n = 6247) had a lower PCS score compared with persons without migration background (−0.52, *p* = 0.001; Table 3). The MCS score was higher (+1.11, *p* < 0.001). The differences in PCS and MCS scores of persons with indirect migration background compared with persons without migration background had *p* = 0.305 and *p* = 0.072, respectively (+0.19 and +0.41, respectively). Mean PCS and MCS scores by sociodemographic characteristics by migration background are shown in Tables S1 and S2 in the online Supplementary Materials.

## 4. Discussion

### 4.1. Main Findings

The aim of this study was to analyze the associations between migration background and physical and mental HrQoL. Mental HrQoL was higher, and physical HrQoL was lower among persons with direct migration background compared with persons without migration background in Germany. No differences in HrQoL were observed between persons with indirect and without migration background.

Direct comparison of HrQoL of persons with migration background and persons without migration background based on samples from the German general population might be biased, because persons with migration background are younger, less likely to be female, more likely to be single, and more often unemployed [30,31]. In order to reduce the imbalance in sociodemographic characteristics in samples from general populations, it is possible to either control for imbalances by using multiple regression models or reweight parts of the samples using propensity score matching methods or entropy balancing [27]. To date, sample-reweighting techniques have been applied only rarely in HrQoL studies based on population surveys. In the last decade, European studies based on National Health and Wellness Surveys used propensity score matching to compare persons with a certain disease with controls without this disease in order to estimate HrQoL differences [32,33,34]. Previous studies that analyzed the association of HrQoL and migration background in Germany used regression analysis to control for imbalances in sociodemographic characteristics [14,16,17]. In the current study, the reweighting of non-migrant comparison samples by entropy balancing resulted in a balance in sociodemographic characteristics. Pre-balancing, the persons of the migrant samples were younger compared with those of the non-migrant sample. Also, martial and employment statuses were differently distributed over migrant and non-migrant samples. Thus, reweighting the comparison samples for imbalances in sociodemographic characteristics was necessary to avoid bias in HrQoL differences, as older age, unemployment, and having never been married, separated, or divorced were found to be associated with lower HrQoL [35]. By employment of the entropy balancing weights in the explanatory models, differences in physical and mental HrQoL between persons with and without migration background can be regarded as unbiased, at least with respect to specific sociodemographic characteristics. Thereby, the weights also take into account differences in the variances of physical and mental HrQoL between the migrant and non-migrant groups. Furthermore, compared to other preprocessing techniques, valuable information is retained from the data, as no information has been discarded by non-matching [27].

Compared with an unbalanced German representative normative sample that has been used to compute SF-12 summary scores, persons with migration background had a higher physical HrQoL (51.5 vs. 50.0) [23]. The mental HrQoL of persons with migration background was also higher compared with that of the German normative sample (50.9 vs. 50.0). As the current sample of persons with migration background and the normative sample were not balanced with respect to sociodemographic characteristics, the difference in physical HrQoL might be explained by the younger age of persons with migration background compared with the persons of the normative sample (39 vs. 48 years) and the negative association between older age and physical HrQoL.

### 4.2. Previous Research and Possible Explanations

Earlier studies from Germany that were based on cross-sectional and longitudinal data found inconclusive results concerning HrQoL of migrant populations. Concerning physical HrQoL, in one sample that was based on longitudinal data of the SOEP, baseline physical HrQoL was higher for persons with direct migration background compared with persons without migration background [16]. The current study and two other studies, however, found lower physical HrQoL for persons with direct migration background compared with persons without migration background [14,36]. One representative population-based study found no difference in physical HrQoL between persons with direct migration background and persons without migration background [17]. No study, included the current study, found any association between physical HrQoL and indirect migration background [14,16,17,36]. Earlier statements concerning different physical HrQoL of migrant and non-migrant samples contained adverse employment situations of migrants and non-migrants as a possible explanation for the difference [16,37]. However, in the current sample, the difference in physical HrQoL persisted after balancing out differences in employment status between non-migrant and migrant samples, indicating another (unobserved) reason for this difference in physical HrQoL.

Concerning mental HrQoL, one study that used a chain sampling technique found lower mental HrQoL for persons with Polish migration background compared with persons without migration background, whereas the current study found higher mental HrQoL for persons with direct migration background compared with persons without migration background [36]. Other studies did not find any associations between mental HrQoL and direct migration background [14,16,17]. In the sample that was based on longitudinal data of the SOEP, a higher baseline mental HrQoL was found for persons with indirect migration background compared with persons without migration background [16], whereas no difference was found in the current study or in any of the other studies [14,17,36].

All this amounts to the fact that the current generation of persons with migration background who had immigrated to Germany during the years 1994 to 2013 still have a lower physical HrQoL compared with persons without migration background. In addition, those persons with migration background seem to be better off with respect to mental HrQoL compared with persons without migration background and probably also compared with older migrant generations. A possible explanation for the difference in physical and mental HrQoL might be that there is a certain probability of interpreting the meaning and answering items of the SF-12 in a different way by persons with different nationalities [38,39]. Furthermore, the lower physical HrQoL might be affected by unknown migration-specific characteristics or an inadequate access to healthcare services for persons with migration background [40]. Finally, there is a distinct need for investigation of the reasons of the better mental HrQoL of persons with migration background and subsequently their reinforcement.

### 4.3. Generalizability

The data of the SOEP used in the current study were representative of German households. The additional two migrant samples M1 and M2 that were integrated into the SOEP, however, were selected to represent the countries of origin of migrants which recently became increasingly important; thus, certain migrant groups were overrepresented [21]. Groups of countries from new Eastern European Union and southern European Union member states and Arab and Islamic countries were overrepresented in the sample, as immigration from those countries increased significantly in the last decade. Furthermore, households of the so-called guest workers were overrepresented in order to represent their descendants better in the SOEP. In 2017, about 23.6% of the total population had a migration background in Germany [1]. This proportion was lower than the proportion of persons with migration background in the sample of the current study (28.8%).

As this study can be considered exploratory, statistical significance of group differences and associations with respect to HrQoL found using statistical tests should be interpreted with caution. According to Wasserstein et al. [41,42], conclusions should not be drawn only on statistical significance in conjunction with arbitrary levels of significance, and differences and associations are neither present nor absent just because of statistical (in)significance. Indeed, the differences and associations with respect to HrQoL should be benchmarked against their real-life relevance. Such a benchmark could be the minimal (clinically) important difference of the PCS and MCS, which were commonly defined to be 3.5 to 5.0 (e.g., [43,44]). With respect to this benchmark, the associations with regard to HrQoL found in the current study should be interpreted restrainedly.

### 4.4. Strengths and Limitations

To our knowledge, this was the first study of HrQoL between persons with migration background and persons without migration background in Germany that used data from the migrant samples from the SOEP. One major strength of this study is the use of a large migrant and non-migrant sample that was based on a representative German household panel.

However, this study also has some limitations. First, the analyses of this study were based on cross-sectional data, and therefore information on the temporal ordering of causes and effects was not evaluated. Furthermore, only data sets from the years 2014 and 2016 were available at the time of the analysis. However, as the aim of the study was to explore associations between migration background and the physical and mental HrQoL, the recency of data was not a truly decisive factor. Future confirmatory studies should nevertheless include also forthcoming SOEP data sets and preferably analyze the data longitudinally. Second, better integrated and more highly educated persons with migration background, i.e., with better German language skills, might have been included in the migrant samples of the SOEP more probably. In order to reduce this bias, the questionnaires of the SOEP were translated into English, Russian, Turkish, Polish and Romanian, and the option of taking an interpreter was given for the interviews [21]. Furthermore, by using entropy balancing as a sample-reweighting technique, the balanced sample of persons without migration background was similar to the samples of persons with migration background with respect to specific sociodemographic characteristics. Nevertheless, the results of this study might not be generalizable to all persons with migration background in Germany. Third, as this study primarily aimed to analyze associations between migration background and HrQoL, no migration-specific characteristics, such as years since arrival in Germany, age at arrival, or citizenship, were considered.

## 5. Conclusions

After the reduction of imbalance in sociodemographic characteristics between the migrant and non-migrant samples, persons with direct migration background had a lower physical HrQoL and a higher mental HrQoL than persons without migration background. It has to be highlighted that persons who are descendants of parents with direct migration background who became sedentary in Germany did not differ with respect to physical and mental HrQoL compared to persons without migration background. Appropriate measures with respect to target-specific healthcare services and clinical implications should be taken by researchers and policy-makers in order to address the reduced physical HrQoL of persons with direct migration background who migrated during the new migratory flows that have emerged in Europe. However, with respect of the exploratory character of this study and the doubtful benchmark of those associations, such advice should be adopted with caution. Notwithstanding, further research is needed in order to determine the causalities behind the lower physical HrQoL and the higher mental HrQoL of persons with direct migration in Germany.

## Figures and Tables

**Table 1:** Sociodemographic characteristics of the sample, pre-balancing (survey years 2014 and 2016).

Sociodemographic Characteristic	Persons without Migration Background Pre-Balancing (n = 21,109)	Persons with Migration Background (n = 8533)	Persons with Direct Migration Background (n = 6247)	Persons with Indirect Migration Background (n = 2286)
Age: Mean (SE)	49.55 (0.12) **	38.73 (0.16) **	42.21 (0.18) **	29.25 (0.22) **
Sex: N (%)				
Female	11,370 (53.86)	4580 (53.67)	3383 (54.15)	1197 (52.36)
Male	9739 (46.14)	3953 (46.33)	2864 (45.85)	1089 (47.64)
Grouped age: N (%)				
18–29	3200 (15.16) **	2438 (28.57) **	1129 (18.07) **	1309 (57.26) **
30–39	3066 (14.52)	2454 (28.76)	1856 (29.71)	598 (26.16)
40–49	4642 (21.99)	1885 (22.09)	1620 (25.93)	265 (11.59)
≥50	10,201 (48.33)	1756 (20.58)	1642 (26.28)	114 (4.99)
Marital status: N (%)				
Never married/single	5210 (24.68) **	2638 (30.92) **	1210 (19.37) **	1428 (62.47) **
Married/in partnership	12,009 (56.89)	5038 (59.04)	4315 (69.07)	723 (31.63)
Separated/divorced	2611 (12.37)	692 (8.11)	568 (9.09)	124 (5.42)
Widowed	1279 (6.06)	165 (1.93)	154 (2.47)	11 (0.48)
Employment status: N (%)				
Employed fulltime	7908 (37.46) **	3207 (37.58) **	2444 (39.12) **	763 (33.38) **
Employed part-time	3098 (14.68)	1070 (12.54)	838 (13.41)	232 (10.15)
Apprenticeship	573 (2.71)	382 (4.48)	151 (2.42)	231 (10.10)
Marginally employed	1348 (6.39)	747 (8.75)	526 (8.42)	221 (9.67)
Unemployed	8182 (38.76)	3127 (36.65)	2288 (36.63)	839 (36.70)
Nationality ^1^: N (%)				
German	21,109 (100.00) **	4554 (53.37) **	2798 (44.79) **	1756 (76.82) **
East European	-	1139 (13.35)	1131 (18.10)	8 (0.35)
South European	-	1311 (15.36)	1015 (16.25)	296 (12.95)
West and North European ^2^	-	284 (3.33)	260 (4.16)	24 (1.05)
African	-	125 (1.46)	121 (1.94)	4 (0.17)
Asian	-	1004 (11.77)	814 (13.03)	190 (8.31)
American/Oceanian	-	98 (1.12)	91 (1.46)	7 (0.31)
Stateless	-	18 (0.21)	17 (0.27)	1 (0.04)

Comments: SE: Standard error; comparison of mean age of persons with and without migration background was analyzed using Student’s *t*-test; comparison of categorical characteristics of persons with and without migration background was analyzed using Pearson’s chi² test; comparison of mean age of persons with direct and indirect migration background was analyzed using Student’s *t*-test; comparison of categorical characteristics of persons with direct and indirect migration background was analyzed using Pearson’s chi² test; ^1^ Nationality was not considered for balancing; ^2^ Without German nationality; ** *p* ≤ 0.001.

**Table 2:** Mean PCS and MCS scores by sociodemographic characteristics and migration background (survey years 2014 and 2016).

Sociodemographic Characteristic	Mean PCS (SE)		Mean MCS (SE)	
	Persons without Migration Background (Balanced Sample; n = 21,109)	Persons with Migration Background (n = 8533)	Persons without Migration Background (Balanced Sample; n = 21,109)	Persons with Migration Background (n = 8533)
Total sample	51.87 (0.08)	51.52 (0.11)	49.96 (0.09) **	50.87 (0.10) **
Sex				
Female	51.57 (0.12) **	50.95 (0.15) **	48.86 (0.13) **	49.76 (0.14) **
Male	52.22 (0.12)	52.19 (0.15)	51.24 (0.13) **	52.15 (0.15) **
Grouped age				
18–29	55.28 (0.15)	55.35 (0.14)	49.60 (0.20) **	50.99 (0.19) **
30–39	53.19 (0.18)	53.51 (0.17)	49.51 (0.20) **	50.71 (0.19) **
40–49	51.20 (0.15)	50.79 (0.22)	49.92 (0.16)	50.56 (0.23)
≥50	46.03 (0.12) **	44.22 (0.26) **	51.18 (0.12)	51.24 (0.25)
Marital status				
Never married/single	54.58 (0.13)	55.09 (0.15)	49.60 (0.16) **	50.73 (0.18) **
Married/in partnership	51.12 (0.11) **	50.40 (0.14) **	50.46 (0.12) **	51.32 (0.13) **
Separated/divorced	48.91 (0.27)	48.18 (0.41)	47.61 (0.30)	48.63 (0.43)
Widowed	44.00 (0.14)	42.73 (0.91)	50.42 (0.48)	48.77 (0.91)
Employment status				
Employed fulltime	53.02 (0.11)	53.16 (0.14)	50.73 (0.12) **	52.14 (0.15) **
Employed part-time	52.45 (0.17)	51.67 (0.28)	49.94 (0.19)	50.34 (0.29)
Apprenticeship	54.94 (0.29)	55.04 (0.36)	50.90 (0.41)	50.65 (0.47)
Marginally employed	52.56 (0.29)	52.16 (0.33)	48.82 (0.35)	49.99 (0.33)
Unemployed	49.96 (0.17)	49.21 (0.20)	49.34 (0.18)	49.98 (0.19)
Nationality				
German	51.87 (0.08)	51.60 (0.14)	49.96 (0.09)	50.42 (0.14)
East European	-	52.39 (0.26)	-	52.63 (0.26)
South European	-	51.39 (0.27)	-	51.13 (0.26)
West and North European ^1^	-	51.47 (0.57)	-	51.07 (0.60)
African	-	52.20 (0.84)	-	50.82 (0.76)
Asian	-	50.16 (0.33)	-	50.62 (0.31)
American/Oceanian	-	53.41 (0.93)	-	50.52 (0.93)
Stateless	-	47.34 (2.52)	-	48.12 (2.39)

PCS: Physical Component Summary; MCS: Mental Component Summary; SE: standard error; comparison of mean PCS and MCS scores by migration background were analyzed using Student’s *t*-test; ^1^ without German nationality; ** *p* ≤ 0.001.

**Table 3:** Differences in PCS and MCS scores by sociodemographic characteristics between persons with direct/indirect and without migration background (survey years 2014 and 2016).

Sociodemographic Characteristic	Mean Diff. ^1^ in PCS (SE)		Mean Diff. ^1^ in MCS (SE)	
	Persons with Direct Migration Background (n = 6247)	Persons with Indirect Migration Background (n = 2286)	Persons with Direct Migration Background (n = 6247)	Persons with Indirect Migration Background (n = 2286)
Total sample	−0.52 (0.16) **	0.19 (0.19)	1.11 (0.16) **	0.41 (0.23)
Sex				
Female	−0.84 (0.22) **	−0.05 (0.28)	1.12 (0.22) **	0.37 (0.32)
Male	−0.15 (0.22)	0.45 (0.25)	1.10 (0.22) **	0.47 (0.32)
Grouped age				
18–29	0.06 (0.28)	−0.01 (0.23)	2.05 (0.35) **	0.91 (0.32) *
30–39	0.33 (0.27)	0.39 (0.36)	1.75 (0.31) **	−0.48 (0.43)
40–49	−0.58 (0.30)	1.08 (0.56)	0.71 (0.30)	0.05 (0.63)
≥50	−1.82 (0.30) **	−0.70 (1.04)	0.11 (0.29)	0.30 (0.90)
Marital status				
Never married/single	0.40 (0.29)	0.40 (0.21)	1.61 (0.32) **	0.79 (0.30)
Married/in partnership	−0.76 (0.19) **	0.16 (0.35)	1.05 (0.19) **	−0.29 (0.37)
Separated/divorced	−0.47 (0.54)	−1.79 (1.04)	1.30 (0.57)	0.10 (1.09)
Widowed	−1.18 (1.06)	−2.45 (3.52)	−1.97 (1.08)	1.97 (2.70)
Employment status				
Employed fulltime	0.06 (0.20)	0.28 (0.29)	1.67 (0.22) **	0.61 (0.36)
Employed part-time	−0.90 (0.36)	−0.39 (0.63)	0.87 (0.37)	−1.29 (0.69)
Apprenticeship	−0.33 (0.67)	0.31 (0.53)	0.29 (0.88)	−0.50 (0.71)
Marginally employed	−0.55 (0.52)	−0.17 (0.57)	1.19 (0.54)	0.86 (0.72)
Unemployed	−1.02 (0.31) **	0.33 (0.34)	0.64 (0.30)	0.84 (0.42)
Nationality				
German	−1.26 (0.21) **	0.41 (0.21)	0.82 (0.21) **	0.04 (0.26)

PCS: Physical Component Summary; MCS: Mental Component Summary; SE: standard error; comparison of mean PCS scores by migration background were analyzed using Student’s *t*-test; ^1^ mean difference between persons with direct/indirect migration background and persons without migration background (balanced samples); * *p* ≤ 0.004, ** *p* ≤ 0.001.

## Data Availability

The code used during the current study is available from the corresponding author on reasonable request for all interested researchers. Interested parties may contact the Department of Health Economics and Health Services Research, University Medical Center Hamburg-Eppendorf (contact information: Dr. Thomas Grochtdreis, t.grochtdreis@uke.de, +49-40-7410-52405).

## Supplementary Materials

The following are available online at https://www.mdpi.com/article/10.3390/ijerph18073665/s1; Table S1: Mean PCS scores by sociodemographic characteristics by migration background (survey years 2014 and 2016); Table S2: Mean MCS scores by sociodemographic characteristics by migration background (survey years 2014 and 2016).

## References

[1] Statistisches Bundesamt (2018). Statistisches Jahrbuch 2018. Deutschland und Internationales.

[2] SOEP Group (2019). SOEP-Core v34—PPATHL: Person-Related Meta-Dataset. SOEP Survey Papers 762: Series D—Variable Descriptions and Coding.

[3] European Migration Network Second-Generation Migrant. https://ec.europa.eu/home-affairs/what-we-do/networks/european_migration_network/glossary_search/second-generation-migrant_en.

[4] European Migration Network (2021). Migrant. https://ec.europa.eu/home-affairs/what-we-do/networks/european_migration_network/glossary_search/migrant_en.

[5] Razum O., Karrasch L., Spallek J., Razum M.O., Spallek J.P.D.J. (2016). Migration. Bundesgesundheitsblatt Gesundh. Gesundh..

[6] Razum O., Richter M., Hurrelmann K. (2009). Migration, Mortalität und der Healthy-migrant-Effekt. Gesundheitliche Ungleichheit: Grundlagen, Probleme, Perspektiven.

[7] Salt J., Rechel B., Mladovsky P., Devillé W. (2011). Trends in Europe’s international migration. Migration and Health in the European Union.

[8] Nørredam M., Krasnik A., Rechel B., Mladovsky P., Devillé W. (2011). Migrants’ access to health services. Migration and Health in the European Union.

[9] Rechel B., Mladovsky P., Devillé W., Rechel B., Mladovsky P., Devillé W. (2011). Monitoring the health of migrants. Migration and Health in the European Union.

[10] Durieux-Paillard S., Rechel B., Mladovsky P., Devillé W. (2011). Differences in language, religious beliefs and culture: The need for culturally responsive health services. Migration and Health in the European Union.

[11] Führer A., Tiller D., Brzoska P., Korn M., Gröger C., Wienke A. (2019). Health-Related Disparities among Migrant Children at School Entry in Germany. How does the Definition of Migration Status Matter?. Int. J. Environ. Res. Public Health.

[12] Andersen R., Newman J.F. (1973). Societal and Individual Determinants of Medical Care Utilization in the United States. Milbank Mem. Fund Q. Health Soc..

[13] Igel U., Brähler E., Grande G. (2010). Der Einfluss von Diskriminierungserfahrungen auf die Gesundheit von MigrantInnen. Psychiatr. Prax..

[14] Nesterko Y., Braehler E., Grande G., Glaesmer H. (2012). Life satisfaction and health-related quality of life in immigrants and native-born Germans: The role of immigration-related factors. Qual. Life Res..

[15] Razum O., Rohrmann S. (2002). The healthy migrant effect: Role of selection and late entry bias. Das Gesundh..

[16] Nesterko Y., Turrión C.M., Friedrich M., Glaesmer H. (2018). Trajectories of health-related quality of life in immigrants and non-immigrants in Germany: A population-based longitudinal study. Int. J. Public Health.

[17] Glaesmer H., Wittig U., Braehler E., Martin A., Mewes R., Rief W. (2011). Health care utilization among first and second generation immigrants and native-born Germans: A population-based study in Germany. Int. J. Public Health.

[18] Rechel B., Mladovsky P., Devillé W., Rijks B., Petrova-Benedict R., McKee M., Rechel B., Mladovsky P., Devillé W. (2011). Migration and health in the European Union: An introduction. Migration and Health in the European Union.

[19] Ingleby D. (2009). European Research on Migration and Health. Background Paper Developed within the Framework of the IOM Project “Assisting Migrants and Communities (AMAC): Analysis of Social Determinants of Health and Health Inequalities”.

[20] Gushulak B. (2010). Monitoring Migrants’ Health. Health of Migrants—The Way Forward.

[21] Brücker H., Kroh M., Bartsch S., Goebel J., Kühne S., Liebau E., Trübswetter P., Tucci I., Schupp J. (2014). The New IAB-SOEP Migration Sample: An Introduction into the Methodology and the Contents.

[22] Andersen H.H., Mühlbacher A., Nübling M. (2007). Die SOEP-Version des SF 12 als Instrument Gesundheitsökonomischer Analysen.

[23] Schupp J., Wagner G., Nübling M., Andersen H.H., Mühlbacher A. (2007). Computation of Standard Values for Physical and Mental Health Scale Scores Using the SOEP Version of SF12v2. Schmollers Jahrb..

[24] Ware J.J., Kosinski M., Keller S.D. (1996). A 12-Item Short-Form Health Survey: Construction of scales and preliminary tests of reliability and validity. Med. Care.

[25] Nübling M., Andersen H.H., Mühlbacher A. (2006). Entwicklung eines Verfahrens zur Berechnung der Körperlichen und Psychischen Summenskalen auf Basis der SOEP-Version des SF 12 (Algorithmus).

[26] United Nations Statistics Division (2006). Standard Country or Area Codes for Statistical Use (M49).

[27] Hainmueller J. (2012). Entropy Balancing for Causal Effects: A Multivariate Reweighting Method to Produce Balanced Samples in Observational Studies. Political Anal..

[28] Hainmüller J., Xu Y. (2013). Ebalance: A Stata Package for Entropy Balancing. J. Stat. Softw..

[29] Dickhaus T. (2014). Classes of multiple test procedures. Simultaneous Statistical Inference: With Applications in the Life Sciences.

[30] Statistisches Bundesamt (2018). Bevölkerung und Erwerbstätigkeit. Schutzsuchende Ergebnisse des Ausländerzentralregisters 2018.

[31] Statistisches Bundesamt (2019). Statistisches Jahrbuch 2019. Deutschland und Internationales.

[32] daCosta DiBonaventura M., Yuan Y., Wagner J.-S., L’Italien G.J., Lescrauwaet B., Langley P. (2012). The burden of viral hepatitis C in Europe: A propensity analysis of patient outcomes. Eur. J. Gastroenterol. Hepatol..

[33] DiBonaventura M., Richard L., Kumar M., Forsythe A., Flores N.M., Moline M. (2015). The Association between Insomnia and Insomnia Treatment Side Effects on Health Status, Work Productivity, and Healthcare Resource Use. PLoS ONE.

[34] Vo P., Fang J., Bilitou A., Laflamme A.K., Gupta S. (2018). Patients’ perspective on the burden of migraine in Europe: A cross-sectional analysis of survey data in France, Germany, Italy, Spain, and the United Kingdom. J. Headache Pain.

[35] Grochtdreis T., König H.-H., Riedel-Heller S.G., Dams J. (2020). Health-Related Quality of Life of Asylum Seekers and Refugees in Germany: A Cross-Sectional Study with Data from the German Socio-Economic Panel. Appl. Res. Qual. Life.

[36] Morawa E., Erim Y. (2014). Health-related quality of life and sense of coherence among Polish immigrants in Germany and indigenous Poles. Transcult. Psychiatry.

[37] Seifert W. (1997). Occupational and Economic Mobility and Social Integration of Mediterranean Migrants in Germany. Eur. J. Popul..

[38] Gibbons C.J., Skevington S.M., WHOQOL Group (2018). Adjusting for cross-cultural differences in computer-adaptive tests of quality of life. Qual. Life Res..

[39] Benítez-Borrego S., Mancho-Fora N., Farràs-Permanyer L., Urzúa-Morales A., Guàrdia-Olmos J. (2016). Differential Item Functioning of WHOQOL-BREF in nine Iberoamerican countries. Rev. Iberoam. Psicol. Salud.

[40] Razum O., Saß A.-C. (2015). Migration und Gesundheit: Interkulturelle Öffnung bleibt eine Herausforderung. Bundesgesundheitsblatt Gesundheitsforschung Gesundheitsschutz.

[41] Amrhein V., Greenland S., McShane B. (2019). Scientists rise up against statistical significance. Nat. Cell Biol..

[42] Wasserstein R.L., Schirm A.L., Lazar N.A. (2019). Moving to a World Beyond “*p* < 0.05”. Am. Stat..

[43] Fontana M.A., Lyman S., Sarker G.K., Padgett D.E., MacLean C.H. (2019). Can Machine Learning Algorithms Predict Which Patients Will Achieve Minimally Clinically Important Differences from Total Joint Arthroplasty?. Clin. Orthop. Relat. Res..

[44] Busse J.W., Bhandari M., A Einhorn T., Schemitsch E., Heckman J.D., Iii P.T., Leung K.-S., Heels-Ansdell D., Makosso-Kallyth S., Della Rocca G.J. (2016). Re-evaluation of low intensity pulsed ultrasound in treatment of tibial fractures (TRUST): Randomized clinical trial. BMJ.

