# Supplementary material for: Health-Related Quality of Life of Persons with Direct, Indirect and No Migration Background in Germany: A Cross-Sectional Study Based on the German Socio-Economic Panel (SOEP)
Source: Int J Environ Res Public Health. 2021 Apr 1;18(7):3665. doi: 10.3390/ijerph18073665 (PMC8037371; doi:10.3390/ijerph18073665)
Supplement: Supplementary file 1 [file ijerph-18-03665-s001.pdf]

Table S1. Mean PCS scores by sociodemographic characteristics by migration background (survey years 2014 and 2016).

Sociodemographic characteristic	Post-balancing sample of persons without migration background for persons with direct migration background (n = 21,109)	Direct migration background (n = 6247)	Post-balancing sample of persons without migration background for persons with indirect migration background (n = 21,109)	Indirect migration background (n = 2286)
	**Mean (SE)**	**Mean (SE)**	**Mean (SE)**	**Mean (SE)**
Total sample	51.02 (0.09)**	50.50 (0.13)**	54.13 (0.09)	54.32 (0.17)
Sex				
Female	50.85 (0.13)**	50.02 (0.18)**	53.65 (0.12)	53.60 (0.25)
Male	51.22 (0.13)	51.07 (0.18)	54.67 (0.12)	55.12 (0.22)
Grouped age				
18–29	55.11 (0.18)	55.16 (0.22)	55.52 (0.13)	55.51 (0.19)
30–39	53.07 (0.19)	53.40 (0.19)	53.47 (0.16)	53.86 (0.33)
40–49	51.07 (0.17)	50.49 (0.24)	51.52 (0.14)	52.60 (0.55)
≥50	45.83 (0.13)**	44.02 (0.26)**	47.83 (0.14)	47.13 (1.03)
Marital status				
Never married/single	54.03 (0.15)	54.43 (0.24)	55.24 (0.12)	55.64 (0.18)
Married/in partnership	50.76 (0.12)**	50.00 (0.15)**	52.67 (0.13)	52.82 (0.32)
Separated/divorced	48.56 (0.29)	48.09 (0.45)	50.37 (0.30)	48.58 (0.99)
Widowed	43.67 (0.47)	42.48 (0.95)	48.66 (0.66)	46.21 (3.45)
Employment status				
Employed fulltime	52.74 (0.11)	52.80 (0.17)	54.02 (0.11)	54.31 (0.27)
Employed part-time	52.29 (0.18)	51.39 (0.31)	53.06 (0.20)	52.67 (0.60)
Apprenticeship	54.86 (0.34)	54.53 (0.58)	55.06 (0.26)	55.37 (0.46)
Marginally employed	51.75 (0.31)	51.20 (0.42)	54.63 (0.30)	54.46 (0.48)
Unemployed	48.30 (0.19)**	47.28 (0.24)**	54.14 (0.17)	54.48 (0.29)
Nationality				
German	51.02 (0.09)**	49.76 (0.19)**	54.13 (0.09)	54.54 (0.19)
East European	-	52.38 (0.26)	-	54.73 (2.65)
South European	-	50.66 (0.32)	-	53.92 (0.46)
West and North European[1]	-	51.35 (0.60)	-	52.75 (1.97)
African	-	52.34 (0.85)	-	47.71 (5.76)
Asian	-	49.44 (0.37)	-	53.24 (0.60)
American/Oceanian	-	53.35 (0.98)	-	54.22 (3.01)
Stateless	-	46.60 (2.64)	-	60.02 (-)

PCS: Physical Component Summary; SE: standard error; comparison of mean PCS scores by migration background were analyzed using Student's t-test; [1] Without German nationality; ** $p \leq 0.001$

Table S2. Mean MCS scores by sociodemographic characteristics by migration background (survey years 2014 and 2016).

Sociodemographic characteristic	Post-balancing sample of persons without migration background for persons with direct migration background (n = 21,109)	Direct migration background (n = 6247)	Post-balancing sample of persons without migration back-ground for persons with indirect migration background (n = 21,109)	Indirect migration background (n = 2286)
	**Mean (SE)**	**Mean (SE)**	**Mean (SE)**	**Mean (SE)**
Total sample	50.05 (0.10)**	51.16 (0.12)**	49.65 (0.11)	50.07 (0.20)
Sex				
Female	49.03 (0.14)**	50.15 (0.17)**	48.30 (0.15)	48.66 (0.28)
Male	51.25 (0.14)**	52.35 (0.17)**	51.14 (0.16)	51.61 (0.28)
Grouped age				
18–29	49.49 (0.24)**	51.54 (0.26)**	49.61 (0.17)**	50.52 (0.26)**
30–39	49.51 (0.23)**	51.26 (0.21)**	49.50 (0.18)	49.01 (0.40)
40–49	49.97 (0.17)	50.68 (0.24)	49.80 (0.15)	49.85 (0.61)
≥50	51.16 (0.13)	51.27 (0.26)	50.57 (0.14)	50.87 (0.89)
Marital status				
Never married/single	49.47 (0.18)**	51.10 (0.26)**	49.63 (0.16)	50.41 (0.25)
Married/in partnership	50.51 (0.12)**	51.57 (0.14)**	50.12 (0.15)	49.83 (0.35)
Separated/divorced	47.59 (0.33)	48.89 (0.47)	47.33 (0.35)	47.43 (1.03)
Widowed	50.63 (0.50)	48.65 (0.96)	48.36 (1.19)	50.33 (2.43)
Employment status				
Employed fulltime	50.85 (0.13)**	52.52 (0.17)**	50.32 (0.15)	50.93 (0.33)
Employed part-time	50.06 (0.19)	50.93 (0.32)	49.50 (0.23)	48.20 (0.65)
Apprenticeship	50.69 (0.46)	50.98 (0.75)	50.93 (0.37)	50.43 (0.60)
Marginally employed	48.97 (0.36)	50.17 (0.40)	48.71 (0.40)	49.57 (0.60)
Unemployed	49.40 (0.20)	50.03 (0.22)	48.98 (0.22)	49.82 (0.35)
Nationality				
German	50.05 (0.10)**	50.87 (0.18)**	49.65 (0.11)	49.70 (0.23)
East European	-	52.63 (0.26)	-	51.93 (3.55)
South European	-	50.95 (0.30)	-	51.68 (0.52)
West and North European[1]	-	51.26 (0.63)	-	48.97 (2.09)
African	-	50.92 (0.78)	-	48.02 (3.14)
Asian	-	50.54 (0.35)	-	50.98 (0.65)
American/Oceanian	-	50.42 (0.99)	-	51.76 (2.19)
Stateless	-	47.46 (2.51)	-	59.35 (-)

Comparison of mean MCS scores by migration background were analyzed using Student's t-test; [1] Without German nationality; ** $p \leq 0.001$
